# Active control of properties of concrete: a (p)review

**DOI:** 10.1617/s11527-018-1256-2

**Published:** 2018-09-20

**Authors:** Geert De Schutter, Karel Lesage

**Affiliations:** Magnel Laboratory for Concrete Research, Department of Structural Engineering, Faculty of Engineering and Architecture, Ghent University, Ghent, Belgium

**Keywords:** Concrete, Active control, Functional polymers, Rheology, Casting, 3D printing

## Abstract

Concrete properties to a large extent depend on mix design and processing, currently leaving only limited options to actively modify concrete properties during or after casting. This paper gives a (p)review on a more advanced active control of properties of concrete, based on the application of external signals to trigger an intended response in the material, either in fresh or hardened state. Current practices in concrete industry that could be considered as active control are briefly summarized. More advanced active control mechanisms as studied in other fields, e.g. based on hydrogels and other functional polymers, are reviewed and some principles are listed. A specific focus is further given on potential methods for active rheology control. Based on the concepts developed in other fields, substantial progress could be made in order to achieve active control of fresh and hardened concrete properties. However, several challenges remain, like the stability and functioning of the responsive material in a cementitious environment, the applicability of the control signal in a cementitious material, and the economy, logistics and safety of a control system on a construction site or in precast industry. Finding solutions to these challenges will lead to marvelous opportunities in general, and for 3D and even 4D printing more particularly.

## Introduction

The quality and performance of concrete as a construction material depend on mix design, processing and casting. The type, quality and dosage of constituent materials are key elements in view of obtaining the right concrete for the right job. Scientific and technological knowledge concerning the properties of concrete [1], supplemented by long-term practical experience, enable to properly design concrete for a given application, either by relying on prescriptive rules as given in many concrete standards, either by aiming for absolute performance further validated by tests [2, 3]. General concrete mix design methodologies are available to design concrete compositions based on the properties of the constituent materials, and aiming for specific applications requiring well-defined physical, chemical and/or mechanical properties.

In addition to the mix design, the material properties will be influenced by the mixing process and the casting operation. Mixing procedure and intensity can significantly influence the properties of the fresh material [4, 5]. Casting operations, possibly including high-pressure and/or long-distance pumping, can further influence the material properties [6]. Obvious influences of processing further include (lack of) vibration and (lack of) curing. When execution of casting operations is properly done according to relevant state-of-the-art prescriptions, the aimed-for concrete properties as considered in the mix design, will normally be achieved.

However, many variations, intentionally or not, play an additional role, sometimes making it a hard challenge to achieve intended concrete properties in fresh and hardened state. As the properties of the constituent materials vary in-between deliveries, maintaining constant concrete quality in spite of the changing constituent properties is not always straightforward. This is illustrated in [7] by studying the effect of variable foaming potential of admixtures on the rheological behaviour of paste. Even with identical constituent materials, small variations in mixing and processing can have noticeable effects. A well-known aspect is the influence of variable moisture content and water addition during mixing. In some cases, e.g. some self-compacting concrete mixtures, small variations in water content can invoke unacceptable variations in workability, making the concrete non-robust [8, 9]. As illustrated in [10], inappropriate rheological properties of the fresh concrete can cause unwanted phenomena like segregation, lack of pumpability, increased formwork pressure, insufficient quality of the surface finish… Undesired extra water addition, excessive air inclusion, insufficient vibration during casting etc. will all have a negative impact on the final mechanical and durability performance of the concrete structure.

Quality control consequently is an important aspect in concrete industry, involving quality control of the constituent materials, quality control of the mixing process, quality control during execution, and quality control (compliance testing) of the final properties. While the quality control actions can be very active in the sense that a lot of people spend a lot of energy in getting things done in the right way, we typically passively rely on the behaviour of the concrete. Once the concrete is mixed and flowing in the pumping pipes or formworks, we have no active methods to change the behaviour of the concrete, e.g. requiring it to flow faster or become stiffer. It is true that pumping pressures could be actively changed, or vibraton methods could be actively modified, but we nevertheless are stuck with a material with a pre-defined rheological response to the external changes. We have no means to modify the material behaviour while processing is ongoing, or when the material is in final position. In other words, we cannot actively change the constitutive behaviour of the concrete, either in fresh or hardened state. We can only change external parameters (e.g. pumping pressure) to invoke an effect (e.g. increase in discharge rate) obeying the constitutive material behaviour.

In other fields, active control methods to change material properties “on demand” do exist. As will be further explained in this paper, special fluids are available responding to an external electric or magnetic trigger signal, changing the behaviour of the material from more fluid-like to more solid-like (or vice versa) on demand [11]. These rheofluids can e.g. be used in smart structures, enabling active intervention for damping of vibrations. Rheological properties like yield stress and viscosity can be actively controlled in a reversible way by applying an external electric or magnetic field. Additional examples of active control of material properties or material actions in other fields will be mentioned further on in this paper, showing very innovative and challenging concepts that will be inspiring for cementitious materials.

Achieving active rheology control (ARC) and active stiffening control (ASC) is the final goal of the ERC (European Research Council) Advanced Grant Project “Smart casting of concrete structures by active control of rheology” (SmartCast) [12]. Active control could also be considered for hardened concrete properties, as will be further illustrated. The concept of active control involves an external action or signal to which the material responds by changing its behaviour. The signal can be given “post-processing”, which means after mixing, during the fresh or hardened state. The concept of active control includes the incorporation of special components or advanced (nano- or micro-) materials into the cementitious material that show a desired response to a given specific signal. In literature, the word ‘control’ is often used, as e.g. in [13] concerning the control of the flow of fresh concrete. Mostly the term ‘control’ in these contexts refers to efficient changes in mixture composition by adding (pre-processing) new or modified components, like e.g. new generation admixtures, in view of obtaining (controlling) desired material properties. Once the cementitious material is mixed, no further intervention is possible to adjust the properties. With active control, we refer to an external signal that can be applied at any time during or after processing of the material, triggering a desired response in the material. The active control can be reversible, or in some cases irreversible.

In this paper, an overview is first given of current practices with active intervention in concrete industry, some of them very traditional, some of them already somewhat pushing the boundaries of current knowledge and understanding. Afterwards, active control mechanisms in other fields are briefly summarized, showing materials, signals and mechanisms. Further focus is then given to methods and applications related to the control of rheological behaviour. Finally, some specific challenges are discussed when transferring existing technologies from other fields to concrete industry in view of enabling active rheology and stiffening control of cementitious materials.

## Current practices with active intervention to control concrete properties

In current concrete practice, active interventions are manifold, although mostly not in the sense of actively modifying material behaviour by triggering a response of mixed-in special components. Nevertheless, an overview of current active intervention methods (as also schematically summarized in Table 1) is a good starting point to learn from, and to push the limit of our current abilities to control fresh and hardened concrete properties.

**Table 1:** Overview of current practices with active intervention to control concrete properties

Type of intervention	“Signal”	“Responsive” constituents	Effect
Mechanical	Vibration (frequency, amplitude, range)	Solid particles (vibration)	Elimination of yield stress (rheology control)
Chemical	Chemical trigger, e.g. retarder, accelerator, CO_2_ …	Cement particles, molecules in pore solution …	Retardation or acceleration of hydration reaction … (setting and hardening control)
Thermal	Temperature field	All constituents (through their thermal properties), cement particles (activation energy)	Control of temperature gradients, control of reaction rate
Hygral	Moisture	Pore system (capillary pressure, diffusion …), Cement particles (reactivity)	Control of shrinkage (cracking), control of hydration process
Pressure	Reduced or increased pressure, or ultrasound	Air	Control of compaction, contribution to accelerated curing, dispersion
Magnetic	Magnetic field	Magnetizable materials (MNPs, steel strips or fibres…), water	Set-on-demand, micro-vibration, effect of magnetized water on hydration, healing on demand …
Electric	Electric current or potential	Dipolar molecules, ions	Control of reaction rate (via heating due to resistivity), formwork release, improved pumping (via polarization)
Microwave	High frequency electric signal	Dipolar elements	Control of reaction rate (via heating due to internal dipolar vibration)

### Mechanical intervention

The direct contact between (aggregate) particles leads to high friction, obstructing the flow. In fresh state, an obvious active mechanical intervention is given by the vibration process, using poker vibration or other techniques. By means of a poker, a vibration with a certain frequency and amplitude is (in this case internally) imposed to the concrete. This “signal” has a certain range of action, triggering the (solid) particles in the cementitious material to respond (vibrate). As a consequence, the yield stress drops to zero within the affected zone [14], and the material liquefies and fills the formwork in a better way (i.e. compacts). While a poker requires an invasive action (‘internal vibration’), other vibration methods are available operating from the surface of the concrete, e.g. formwork vibrators. External vibrators on pumping pipes are sometimes used at regular distances in case of pumping of concrete for tall towers, several hundreds of meters upwards.

Vibration of fresh concrete can thus be seen as the active application of an external signal (mechanical vibration with a certain frequency and amplitude), triggering a response from some constituents in the bulk of the concrete (in this case vibration of solid particles), changing the rheological properties of the fresh concrete (reducing the yield stress to zero). In this way, it could be said that active rheology control of fresh concrete is already performed in concrete industry since the 1930s due to the introduction of poker vibrators. However, active rheology control can go much further, as will be illustrated further on in this paper.

### Chemical intervention

An active chemical intervention is often applied in case of sprayed concrete, introducing accelerating admixtures in the spraying head. The added accelerating admixture triggers the cement particles, speeding up the hydration process in a chemical way, leading to faster setting (stiffening) and hardening of the sprayed concrete. Similar but even more complex chemical interventions can be applied when at the end of a working day the concrete within a long pumping pipe (e.g. for the construction of a tall tower) is removed for pipe cleaning. The concrete flowing back in a truck mixer can be retarded for a long night by adding a chemical retarder. The next morning, while resuming pumping operations, a chemical accelerator can be added, counteracting the effect of the retarder. This kind of active combination of retarders and accelerators has e.g. been studied by Lesage [15].

The active intervention in this case is giving a chemical signal to the cement particles, leading to a control of the hydration rate. After addition of the accelerator the triggered response of the cement particles is irreversible. However, the triggered response of the added retarder can be reversed by later addition of an accelerator. This kind of active intervention is limited to certain moments in the process, e.g. when the concrete is flowing through the spray head in case of sprayed concrete, or when the concrete is in the truck mixer in the case of re-use of concrete in a pumping pipe. The control is no longer possible at other timings, and it is also not possible to adjust the control levels once the admixtures have been added.

Another example of active chemical intervention is the use of carbon dioxide for the accelerated curing of cementitious materials, often in combination with the application of a higher temperature. This active chemical intervention has already been reported by Klemm and Berger [16], but has been given renewed attention in recent years because of environmental issues, as illustrated by Monkman and MacDonald [17], studying the addition of carbon dioxide gas during mixing of the concrete for the production of blocks, and by Nielsen et al. [18], studying carbonate-bonded construction materials from alkaline residues.

### Thermal intervention

The effect of temperature on the hydration process of cement is well-known, and can be calculated by means of the Arrhenius equation [19], with the activation energy as most important parameter. This knowledge is applied in industry by means of heat curing, e.g. steam cycles in precast industry [20, 21]. This involves an active intervention, applying a thermal signal to the hardening concrete in view of accelerating the hydration process and achieving faster setting and strength development (at the cost of reducing somewhat the final strength [22]). The applied thermal field is triggering the cement particles (and/or supplementary cementitious materials) that are responding according to their activation energy, resulting in an acceleration of the hydration process. Steam curing (and heat curing more generally) can be considered as a type of active setting and hardening control.

Another type of active control involving a thermal field is given by cooling measures to mitigate thermal cracking in hardening massive concrete elements [23]. In embedded cooling pipes, a cooling agent is circulated through the massive element, imposing lower temperatures inside the hardening concrete [24, 25]. All constituents of the concrete respond to the applied thermal signal (lower temperature in embedded cooling pipes) following the physical laws of thermal diffusion, as described by Fourier’s equation [26]. Important parameters in this process are the specific heat and the thermal diffusivity of the hardening concrete, as well as the heat production due to the exothermal hydration process [27], also responsive to temperature following the previously mentioned Arrhenius Equation.

### Hygral intervention

Freshly cast concrete needs to be protected against premature drying, in view of mitigating shrinkage cracking and creating optimal conditions for the hydration process. For onsite casting, protection against premature drying during the first days is for many elements achieved in a passive way by the formworks. After striking of formworks, or for those elements not protected by formworks (e.g. slabs), curing can be performed in an active way by water spraying or other techniques. The active intervention providing sufficient moisture near the concrete surface is interacting with the pore system, based on capillary forces and diffusion processes. As a result, the concrete is maintaining sufficient moisture conditions, mitigating or reducing early age shrinkage cracking, and improving the hydration process [28].

Modern concepts of internal curing by means of porous aggregate particles or super absorbent polymers (SAP) [29] cannot be considered as active interventions, as the water contained in those materials is only released internally as a result of the self-desiccation linked to the hydration process. In future, however, active intervention could be envisioned, by making SAPs or similar products to release their water content on demand by providing an external trigger. The triggered release of agents contained in hydrogel-type polymers is a known concept in other fields already now, as will be illustrated further on in this paper.

### Intervention by pressure

Concrete also includes air, showing a potential effect on the rheological properties depending on the size of air bubbles and the applied shear stresses [30]. During processing, air can influence the rheological behaviour, and vice versa, the processing can influence the air system of the concrete [6]. During mixing, pressures in the mixing pan can be reduced, resulting in vacuum mixing [31]. As a result, the air void system of the fresh (and later on hardened) concrete is influenced, resulting in reduced porosity and increased mechanical and durability performance. Modifying the air content in fresh concrete also influences the rheological behaviour [30]. Others made use of frequently changing pressure waves by means of an inline ultrasound application [32]. A process referred to as cavitational shear disperses cement grains better and thereby reduces the setting time and admixture use. This pre-cast method allows to speed up stiffening and is preferentially applied to neat cement paste, before mixing it as concrete. Vacuum conditions can also be applied during casting, resulting in vacuum compaction of the fresh concrete [33]. While curing, air pressures could be increased, possibly combined with increased temperature and/or increased CO_2_-content (see also higher), in view of accelerated hardening of the concrete element [34]. External pressure control can thus trigger changes in air content and pore structure, in view of achieving improved material performance.

### Magnetic intervention

Magnetic fields can be relatively easily applied in industry, and can be an element of control during a production process. A magnetic field could be applied to magnetize water before its addition to cementitious materials. A magnetic field changes the physical properties of water, including e.g. specific heat and evaporation rate [35]. Some researchers claim that the use of magnetized water in cementitious materials has an effect on fresh and hardened properties, e.g. Reddy et al. [36]. They observed that the use of magnetized water in concrete resulted in a very significant increase in concrete strength, probably due to the higher surface area of magnetized water. Su and Wu [37] also observed a strength increase when using magnetized water in mortar and concrete containing fly ash. Nevertheless, the use of magnetized water is still open to significant debate, as it is not clear how water could maintain its magnetization long enough after removal of the magnetic field. It has to be further studied whether the reported improvement of properties really is the effect of previous magnetization of water, or rather the effect of other experimental influences that have not been clearly identified.

Soto-Bernal et al. [38] studied the effect of maintained static magnetic fields applied during hardening on the performance of cement paste. They concluded that more CSH is produced, with denser morphology and reduced porosity. It is conjectured that the magnetic field is influencing the hydration process by means of a restructuration process at molecular scale, resulting in microstructural improvement and increased mechanical performance.

The application of magnetic fields to control rheology of cementitious materials containing magnetic particles in real time has been studied by Nair and Ferron [39]. They considered two types of carbonyl iron powder, added to Portland cement paste (water cement ratio 0.4) in dosages up to 10% by mass of cement (corresponding to 4% in volume). Magnetic field with strengths up to 1.0 T have been applied while performing rheological tests on the fresh samples. It is experimentally shown that the rheological behaviour of the paste containing magnetic particles can be actively altered by applying the magnetic field. The effect is depending on the field strength as well as on the dosage of magnetic particles. On the other hand, the authors conclude that the magnetic field does not seem to have a noticeable effect on the morphology and formation of early age hydration product, and does not influence the final compressive strength. This seems to be in contradiction with the previously mentioned conclusions obtained by Soto-Bernal et al. [38], showing that further research is needed to elucidate the effect of magnetic fields on cement hydration and microstructure development. Disregarding the discussion on the effect of magnetic field on hydration, the research of Nair and Ferron [39] is a major step forward towards active rheology control of cementitious materials.

Abavisani et al. [40] also considered magnetic fields to study the possibility to align steel fibres (chips) according to a certain orientation. They conclude that the application of a magnetic field on the fresh steel chip-reinforced concrete facilitates the compaction process, and leads to an increased compressive strength depending on the test direction. Following the optimum direction, the compressive strength increases by more than 17%. Particularly interesting with regard to rheology control is their observation that the vibration of the steel chips in the alternating magnetic field can be considered as a way to obtain *self*-*compactor elements* for concrete.

The same group of authors further studied the effect of alternating magnetic fields and alternating current on the behaviour of reinforced concrete columns [41], considering magnetic fields up to 0.5 T with frequency of 50 Hz, and AC current up to 36 A. The alternating magnetic field facilitated the compaction of the fresh concrete in the reinforced columns due to the triggered vibration of the reinforcing bars.

Applying the magnetic field on the hardened concrete seems to lead to contradictory results, with sometimes an increase [40] and sometimes a decrease [41] in performance. The authors state that the application of magneto-electric fields can be the basis for *real*-*time compressive behaviour controlling* of reinforced concrete elements, even enabling a control of failure mode. However, their claims are based on contradictory results, and are not fundamentally supported by a clear mechanism explaining the potential influence of the (electro-)magnetic field on the material and structural properties. While the principle of active control of concrete properties by magnetic signals has been set forward, it is not fully clear which mechanism could be the basis for the explanation of the reported effect of magnetization on hydration and microstructure development. This will need further research attention, with more rigorous test protocols in order to fully exclude potential influences of other parameters.

On the other hand, it seems clear that mixed-in magnetic particles or magnetizable materials like steel chips as well as preplaced steel rebars are responding to the magnetic signal, with effects on rheology, compactability and even mechanical performance. This opens a window for new developments and applications, possibly involving new types of magnetizable materials [42] or new fields of application like on-demand healing [43, 44]. In some applications, the effect of the magnetic field is triggering its response via another physical mechanism like (micro-)vibration (making the link with Sect. 2.1) or temperature increase (making the link with Sect. 2.3).

### Electric intervention

An electric control signal (AC current) has been mentioned in previous paragraph, where it was combined with the research on the magnetic control of the mechanical performance of hardened reinforced concrete columns. Other literature results are available on the application of electric fields on fresh or hardening concrete. As an example, Kovtun et al. [45] apply direct current for the curing of alkali-activated fly ash concrete. The current serves as an indirect way of heating, while the higher temperature achieved is leading to the accelerated hardening of the material. In their research, Kovtun et al. used a setup in which the direct current, provided through a controllable unit with maximum current 12 A and voltage up to 260 V, was continuously controlled in order to reach a curing temperature of 60 °C in the concrete, with an initial heating rate of 20 °C in order to avoid too fast heating, similar to what is done when applying steam cycles. While the external signal is electric, the physically controlling mechanisms behind the accelerated hardening is the thermal energy. Nevertheless, the response of the alkali activated material to the electric trigger is depending on the activator used, through its influence on the resistivity of the concrete. A similar principle has been followed by Zhang et al. [46, 47] as described in their patents concerning the control of setting time of alkali-activated concrete through the application of an electric field. The electric field only serves as a practical means to control the temperature in the cementitious material, depending on its resistivity.

A more innovative application of an electrical field can be found in the papers of Goudjil et al. [48, 49]. They impose an electric potential on a steel formwork panel, up to a level of 3 V during 10 min, in view of polarizing the concrete layer close to the formwork surface. The applied electric signal triggers a response in the material, resulting in a reduced adhesion between the hardening concrete and the formwork panel. The induced electro-osmotic effect is thus facilitating the formwork release once the concrete has sufficient strength to be self-supporting. In this way, the application of demoulding oils can be avoided.

Inspired by this electrically controlled formwork removal technique, Choi et al. [50] applied a similar polarization technique to improve the flow of concrete in pumping pipes. In a first pilot test, they externally applied an electromagnetic field around steel pumping pipes in order to polarize the material near the pipe surface and improve the formation of the lubrication layer. Although the electromagnetic field applied by Choi et al. indeed seemed to improve the pumping behaviour of the concrete, as experimentally verified by an ultrasound velocity profiling technique, it is important to notice that they did not add any specifically responding material like magnetic particles to the concrete. They merely relied on the response of the classical constituents present in the concrete. Although they mentioned the increased mobility of magnetized water and the results of the pilot test were positive, the fundamental physico-chemical mechanisms behind the success of this technique need further study and elucidation. Understanding this in detail might open the door to the development of new materials that could be mixed-in, strengthening its response triggered by the polarization signal and potentially enabling to better control the response to an intended level.

Another example of electric intervention is the technique of cathodic protection by means of applied current in view of protecting an existing structure from reinforcement corrosion [51]. This technique is becoming more and more popular in concrete practice, and is a nice illustration of currently available active control mechanisms. The fundamental principle of its functioning can be understood by considering a Pourbaix diagram, explaining passive and active states of reinforcing steel in concrete [52].

### Intervention with microwaves

As an extension of electric signals, microwaves can also be mentioned in this overview. Microwaves are electromagnetic waves with high frequencies (300 MHz to 300 GHz) and wavelengths ranging from 1 mm to 1 m. A dielectric material placed in a high frequency electric field will heat up due to internal friction, as the induced vibration of the dipolar molecules is hindered by molecular attraction forces. As already reported by Xuequan et al. [53], the promoted cement hydration can be attributed to the elevated temperature as a result of the exposure of the hardening concrete to microwaves. However, an additional effect seems to be the desiccation of free water prior to setting, resulting in decreased porosity. As a result, microwave curing of concrete yields very high early strength, while also providing slightly higher strength at 7 and 28 days [53, 54].

## Active control in other fields

In other fields, functional materials get a lot of research attention. For a multitude of potential applications, controllable materials are developed, responding to specific trigger signals, and resulting in an aimed-for effect. In this section, an (non-exhaustive) overview is given of some applications, type of materials, type of signals and type of responses. Some specific attention is given to different options to control rheology. The applications and control methods used in other fields can be inspiring in view of reaching active control of properties of concrete.

### Examples of applications

A major field of research studying stimuli-responsive materials is given by *drug delivery on demand* [55–63]. Complex three-dimensional polymer structures (see Sect. 3.2) have been developed that carry drugs or special agents. The polymers are stimuli-responsive, which means that as a result of some trigger signal (see Sect. 3.3) they change their structure or undergo other modifications, resulting in the release of the drug at the intended location in the body. These drug delivery systems have great potential in the fight against cancer and other major diseases. In this context, the development of biosensors can also be mentioned, as they can be integrated within drug delivery systems [64].

Stimuli-responsive polymers are also used for *soft robotics* and *micro*-*actuators*, when the functional polymer can transform chemical energy into a mechanical action like folding or bending [65–69]. Typically, electro-responsive hydrogels are considered for this field of application, as the control is repeatable and reversible. In advanced applications, the stimuli-responsive polymers can be used for the development and control of *self*-*propelled nanomotors* [70].

In this non-exhaustive overview of applications of functional materials, reference can also be made to *adhesion on demand* [71], *smart windows* [72] and even the possibility to write *information* into triggered hydrogel by very precise control of the electric signal given to self-assembling molecules [73].

### Type of polymers

The majority of current applications relying on stimuli-responsive control is based on *hydrogel*, generally defined as *three*-*dimensional network structures consisting of polymeric chains joined by tie points or joints and swollen in water up to thermodynamic equilibrium* [74]. Hydrogels have already been studied for a long time, with early work in 1930s and 1940s [74]. It is a very broad class of materials that can be synthesized in numerous ways in view of obtaining a wide range of properties and behaviour [75]. The concept of hydrogels is based on crosslinking of hydrophilic polymers into a spatial complex structure that is able to swell or de-swell depending on the thermodynamic equilibrium within the surrounding medium. In case of ionic hydrogels, the ionic interactions bring additional forces that influence the swelling equilibrium. A major parameter is the pH-value of the surrounding medium, which in current fields of application is typically much lower than in cementitious materials. Nevertheless, hydrogels can be applied in cementitious materials, as illustrated by the well-known application of SAP [29].

Although gels offer great possibilities, they have limitations concerning multi-responsivity and multi-functionality. An alternative approach is the use of *vitrimers*, covalently crosslinked polymer networks. Vitrimers can be modified in different ways, and even be combined with carbon nanotubes, resulting in many different functionalities, in response to even six different stimuli [76].

A specific group of materials is given by *core*–*shell type particles* with monodisperse polymeric cores and uniform shells with independent control of properties of core and shell [77]. Core–shell microspheres dispersed in oils are a nice example of electrorheological fluids that can be used as e.g. dampers, brakes and shock absorbers.

Stimuli-responsive polymers intended for the (reversible) control of shape are also called *shape memory polymers* (SMP). The mechanism can be based on different controllable transitions, such as reversible molecule cross-linking or anisotropic-isotropic transition. They can be reinforced by different materials, e.g. carbon nanotubes, carbon nanofibers, exfoliated nanoclay…, to improve the mechanical performance of the resulting material, called *shape memory polymer composites* (SMPC) [78]. They could also include Fe_3_O_4_-particles, or other magnetizable materials in order to make them magneto-responsive [79, 80].

Magnetic nanoparticles (MNP) can also be incorporated into *hyperbranched polymers* to make them magneto-responsive. Hyperbranched polymers show good possibilities to make them stimuli-responsive in general, offering good options to include a wide choice of terminal functional groups and branches [81].

The concept and design of stimuli-responsive polymers in general is getting a lot of research attention nowadays. New design strategies [82] and new synthesis and polymerization methods [83] are important aspects in obtaining new stimuli-responsive materials that could also be applicable in cementitious environments, overcoming specific challenges (see Sect. 4) that currently pose problems to most of the existing responsive materials.

### Type of signals

Most common signals concern temperature and pH (or pH difference), or the application of an electric, magnetic, or electromagnetic field. Many other signals, however, have shown to be applicable in view of inducing a triggered response in functional materials, like e.g. pressure [84], redox-signal [55, 64, 85–88], carbon dioxide [85, 89, 90], light [87, 91], ultrasound [58], UV [92–94] …

Most stimuli-responsive polymers respond to one or more trigger signals. In case of dual or multi-responsive materials, the desired action depending on the given combination of trigger signals is an important point of attention. In case of two signals, as an example, it could be that the desired response is only activated in the presence of both stimuli, while another option could be the activation in case of any single stimulus. Different other logic combinations can be chosen, as illustrated in [57].

The applicability of the trigger signal depends on the type of material, and further depends on practical considerations. While for some applications photonic signals are convenient and environmentally friendly, the responsive process (often chemical) is typically not showing a high efficiency [95]. For opaque materials like cementitious materials, light signals are not a good option, unless only a response at the surface is needed. Signals based on change in pH or on electrochemical responses are typically having a higher efficiency. Electric or redox signals further depend on the resistivity or conductivity of the material, and are thus not applicable in all circumstances. Heating and cooling seem easy trigger systems in principle, but are not very practical in many cases.

### Type of responses

Roy et al. [96] give an interesting overview of stimuli-responsive materials, with clear illustrations of their effects. Numerous types of responses, resulting in different effects can be listed. In general, control can be reversible or non-reversible, depending on the type of triggered response and the type of signal. A non-exhaustive list of different types of triggered responses is given hereafter:

- Triggering crosslinking resulting in gel formation, often by means of thermally induced sol–gel transition [97]
- Cleavage of polymer molecules, resulting in collapse of a hydrogel or a hyperbranched polymer, or influencing micellization [58, 91, 94]
- Controlling swelling/de-swelling and the release of an agent, due to changes in osmotic pressure by asymmetric distribution of ions in electric field [65], or by change of electronic charge surface potential [61]
- Changing the permeability of a responsive membrane, by closing or opening “chemical gates”, e.g. by pH control [96]
- Sorption control of water vapour of e.g. electro-responsive polypyrole film [98]
- Control of solubility, often by changing temperature [99]
- Changing the nature of a complex molecule from hydrophilic to hydrophobic, also enabling control over the micellization process [97]
- Changing the self-assembly, e.g. CO_2_ and redox-stimulated transformation of entangled worms to rodlike micelles [85]
- Changing the orientation of the polymers, e.g. by the application of an electric and/or magnetic field [100, 101]
- Controlling entanglement of polymer chains [102]
- Controlling aggregation/dispersion of particles in a medium [92]
- Control of trans–cis isomerization of polymers [93]
- Shape-morphing [103]
- Oscillatory shape change, e.g. oscillatory bending [103, 104]
- Crystallization/melting transition [78]
- Crystal anisotropic/isotropic transition [78]
- …

The listed responses can induce changes in numerous material properties, or can lead to specific actions (like e.g. drug release). Some of the listed responses could have good potential for rheology control. This is further discussed in the following paragraph.

### Existing methodologies for rheology control

The rheological behaviour of a material is governed by fundamental physical properties, characterized by specific parameters, further depending on the type of material [105]. For solid materials, the ordering of crystals (in case of crystalline materials) is an important aspect, or the absence of ordering (in case of amorphous or glassy materials). The deformation behaviour, e.g. characterized by elastic properties, is largely depending on the internal mutual interaction forces between crystals and/or amorphous phases. On the other end of the material spectrum, the deformation behaviour of simple liquids (like water) is mainly governed by the atoms or molecules, responding to Van der Waals forces and thermal agitation, and is e.g. characterized by the viscosity which is the only relevant parameter in case of a Newtonian fluid.

When particles are introduced into a liquid matrix, the behaviour can be different depending on the size and nature of the particles, and the resulting interparticle forces. In suspensions, depending on the density of the particles and of the suspending liquid, particles could segregate down- or upwards, as governed by the interplay between gravitational forces and buoyancy forces. However, particles also interact, depending on their concentration in the suspending liquid, counteracting settlement forces. As a result, the viscosity of the suspension will be different from the viscosity of the suspending medium. For low particle concentrations, up to 30% of the maximum concentration, the viscosity increase is limited. However, at larger concentrations, the viscosity can increase significantly, as described in the Krieger–Dougherty equation or similar models [106]. Furthermore, orientation or alignment of particles can significantly influence the viscosity compared to the case with random orientation of particles.

In case of small particles, in the range of µm and below, colloidal interactions become dominant, mainly Van der Waals forces combined with Brownian motion, and the particles tend to form clusters or aggregates. This aggregation phenomenon could be counteracted by electrostatic repulsion or by steric hindrance, as typically done by superplasticizers in cementitious materials [107]. Depending on the internal structure formation by clustering of particles, the rheological response can be diverse and complex, involving concepts like yield stress (static and dynamic), thixotropy, shear-thickening or shear-thinning…

When the particles in the liquid are polymers, droplets or air bubbles, the overall rheological behaviour of the resulting fluid can become even more complex, with additional governing parameters. Although advanced mathematical models are available [108], based on fundamental principles, the experimental characterization of the complex fluids is not an easy task. Measuring rheological properties is not straightforward [109], and rheological constitutive laws are mostly empirical with limited validity. In many research papers, small amplitude oscillatory shear (SAOS) is applied, resulting in the determination of the so-called storage modulus *G*′ (representing the energy storage or elastic property of the fluid) and the loss modulus *G*″ (representing the viscous dissipation in the fluid) [110]. In case of changing rheological properties of systems with smart functional materials, oscillatory rheometry is an interesting experimental method to study the reversible or non-reversible transition from more liquid-like to more solid-like behaviour.

Considering the governing physics and the resulting characteristic parameters of complex fluids, changing or controlling the rheological behaviour can involve different mechanisms or approaches. A non-exhaustive overview is given hereafter.

A well-known concept to change the rheological properties of a fluid is given by electrorheological (ER) fluids [11, 111, 112] and magnetorheological (MR) fluids [113, 114]. Electrorheological response was first reported by Winslow already in 1949 [115]. In these suspensions, electro- or magneto-responsive particles align along the field lines of the applied electric or magnetic field, forming chain- or column-like structures, changing the rheological properties of the material from liquid-like to solid-like. The transition is reversible, and provides a means to actively control the rheological response of the material, with potential application in many fields of engineering.

In terms of rheological properties, it is reported that the yield stress (*τ*_*y*_) in ER fluids is depending on the electric field strength (*E*_0_) in the following way:

1 $$\tau_{0} \propto E_{0}^{a}$$

with parameter *a* typically in the range of 1.5–2.0 [11]. Instead of applying Bingham-type models, a modified constitutive equation, called the Cho–Choi–Jhon (CCJ) model, is often referred to in case of ER or MR fluids [11, 111, 114]. Due to the formation of an internal structure under electric or magnetic field, the loss modulus *G*″ typically shows an initial plateau as a function of shear stress, while the storage modulus *G*′ is higher than the loss modulus *G*″. In electro- or magneto-activated state, the suspension is dominantly showing an elastic behaviour. However, when the shear stress exceeds a critical value, the storage modulus *G*′ shows a sudden decrease, physically governed by the rupture of the internal structure and the resulting onset of material flow [11, 111, 114]. SAOS can elucidate in this way the internal structure formation in electric or magnetic fields, providing data in view of accurate electro- or magneto-rheological control.

Besides internal structure formation triggered by electric or magnetic field, other mechanisms can be considered for rheology control. One example is based on micro-vibration or micro-actuation. In Sect. 2.6, it was already mentioned that alternating magnetic field applied to cementitious materials containing steel chips can make the chips work as micro-vibrators [40]. The same principle could be applied to alternating field acting on electro-responsive polymers or polymer composites, that could take the role of microvibrators [66–68].

Coming back to some fundamental parameters governing the rheological behaviour of suspensions and colloids, different triggered responses listed in Sect. 3.4. could also be useful in view of controlling rheological behaviour. Hydrogels can be helpful in sol/gel transitions, release of agents potentially modifying viscosity of the suspending medium, swelling/de-swelling mechanisms, controlling micellization, shape morphing… The wide range of potential control mechanisms of hydrogels is offering also many opportunities to control rheology of the complex material containing the hydrogels. It is to be mentioned however, that rheology control by means of hydrogels has not been the main point of attention of hydrogel research up to now, while the focus was mainly on other intended applications, like e.g. drug delivery. Any controlled response influencing interparticle forces, particle clustering, viscosity of the suspending liquid, chemistry of the suspending liquid etc. offers a potential mechanism for rheology control. Some examples where rheology control is at least partly reported can be found in literature, like e.g. capillary oil suspensions containing calcium carbonate particles [116], aggregation in aqueous solutions by working on hydrophobicity and micellization [92], controlled solubility by temperature and photocleavage [94], controlled release of nanoparticles [56].

## Challenges for active control in concrete

From a rheological point of view, concrete is a very complex material. It combines features of a colloid and a suspension, while showing at the same time properties of a granular material. In current approaches, reaching desired workability levels in fresh concrete is typically achieved by appropriate mix design, including the use of superplasticizers working on the principle of electrostatic repulsion or steric hindrance [107]. This, however, is a passive way of rheology control, leaving no possibility to adjust workability levels while the concrete is already flowing in a pumping pipe or in a formwork.

Active control is available in some way, as mentioned in paragraph 2. Considering the numerous control mechanisms studied in other fields, as summarized in general and related to rheology more specifically (paragraph 3.), it is clear that new ways could be found to actively control concrete properties in general, and rheological properties of the fresh concrete more specifically. However, many difficulties have to be overcome when applying control mechanisms from other fields to the field of cementitious materials. The authors selected three concrete characteristics that are forming the main obstacles for now:

- High pH
- Low signal conductivity
- Low signal transmission

### High pH

The fascinating mechanism displayed by different types of hydrogels, as discussed in Sect. 3.2, is tempting to pursue for fresh concrete. However, the existing stimuli responsive hydrogels have been switching between swelling and de-swelling at pH’s that generally occur in the human body, i.e. pH ≤ 7.4. The precarious balance between the fixed charges in the polymer network and the osmotic pressure due to inner mobile ions is sensitive to ion concentration changes in the surrounding fluid. In contrast, a subtle ion concentration change in concrete with a pH of ± 12.5 will not be sensed by available weak polybases (pH 7–11) due to the relative abundance of hydroxyl and other ions. Some applications of hydrogels do exist in concrete industry, like the use of SAP, however, without external control features. They could show some autogenous response, e.g. when SAPs are implemented as a method for self-healing [117]. However, further developments are needed to make them respond to active trigger signals to show on-demand responses.

### Low signal conductivity

The application of control signals in cementitious materials also comes with some challenges. Some signals will not be applicable, like light signals, as they will not be able to travel easily through an opaque cementitious material. Electric control of hydrogels or other functional polymers is often based on an isolating dielectric medium suspending the functional polymers [112]. In contrast, fresh cementitious materials contain water with a relatively high ionic conductivity and cannot be considered electrically isolating.

### Low signal transmission

Another challenge is posed by the large bulk volume and its inert constituents. As an example, while casting a massive foundation slab or a high wall, will it be possible for the trigger signals to reach the bulk of the concrete? With the intended amplitude and/or frequency? In any location? These questions involve different aspects, including fundamental knowledge on the interaction between the physical fields and large volume of concrete in a real environment, involving e.g. pumping pipes and formwork. Furthermore, there are as well practical questions concerning the required installations to provoke the intended fields. Safety issues will become important when working with electromagnetic or other signals on a real construction site.

In some cases, however, bulk control will not be required. As an example, the pumping behaviour to a very large extent depends on the properties of the lubrication layer near the pipe surface [118]. In that case, an economical question might emerge, related to the cost of adding (probably relatively expensive) functional material to the bulk of the concrete, while only needing them for control purposes in a limited zone of interest.

Summarizing the challenges, the following aspects will require due attention when developing active control features for fresh and hardened concrete:

- Stability and functioning of the responsive material in a cementitious environment, typically showing higher pH values than in other fields
- Applicability of the control signal in a cementitious material, depending on its physical properties like resistivity, conductivity, opacity…
- Economy, logistics and safety of a control system on a construction site or in precast industry

In spite of the many challenges, a good opportunity might be given by digital fabrication methods, including 3D printing. Within the relatively limited volume of a printing head, it will be more practical and realistic to provide control signals to modify the rheology of the fresh cementitious material when moving from pumping line to printed layer. As the rheological requirements in a pumping pipe and for a printed layer are significantly different, an active control of the material properties in the printing head would be a very efficient approach. Active control features embedded in 3D printed concrete could open the door to so-called 4D printing in construction industry, as already envisioned by Momeni et al. [119].

## Summary

This paper gives a (p)review on the active control of properties of concrete, based on the application of external signals to trigger an intended response in the material, either in fresh or hardened state. Current practices in concrete industry that could be considered as active control have been listed. More advanced active control mechanisms as studied in other fields, e.g. based on hydrogels and other functional polymers, are reviewed and briefly summarized. A specific focus is further given on potential methods for active rheology control. Based on the concepts developed in other fields, substantial progress could be made in order to achieve active control of fresh and hardened concrete properties. However, several challenges remain, like the stability and functioning of the responsive material in a cementitious environment, the applicability of the control signal in a cementitious material, and the economy, logistics and safety of a control system on a construction site or in precast industry. Finding solutions to these challenges will lead to marvelous opportunities in general, and for 3D and even 4D printing more particularly.
